# Coinfection of HPVs Is Associated with Advanced Stage in Colorectal Cancer Patients from Qatar

**DOI:** 10.3390/pathogens12030424

**Published:** 2023-03-08

**Authors:** Queenie Fernandes, Ishita Gupta, Khaled Murshed, Hayan Abo Samra, Hamda Al-Thawadi, Semir Vranic, Mahir Petkar, Giridhara Rathnaiah Babu, Ala-Eddin Al Moustafa

**Affiliations:** 1 College of Medicine, QU Health, Qatar University, Doha P.O. Box 2713, Qatar; 2 Translational Cancer Research Facility, National Center for Cancer Care and Research, Translational Research Institute, Hamad Medical Corporation, Doha P.O. Box 3050, Qatar; 3 Department of Laboratory Medicine and Pathology, Hamad Medical Corporation, Doha P.O. Box 3050, Qatar; 4 Biomedical Research Center, QU Health, Qatar University, Doha P.O. Box 2713, Qatar; 5 Oncology Department, McGill University, Montreal, QC H3A 0G4, Canada

**Keywords:** colorectal cancer, human papillomavirus, high-risk HPV coinfection, Qatar

## Abstract

High-risk human papillomaviruses (HPVs) are considered risk factors in the origin of several human malignancies, such as breast, cervical, head and neck, as well as colorectal cancers. However, there are no data reported on the HPV status in colorectal cancer in the State of Qatar. Therefore, we herein examined the presence of high-risk HPVs (16, 18, 31, 33, 35, 45, 51, 52, and 59), using polymerase chain reaction (PCR) in a cohort of 100 Qatari colorectal cancer patients, and their association with tumor phenotype. We found that high-risk HPV types 16, 18, 31, 35, 45, 51, 52, and 59 were present in 4, 36, 14, 5, 14, 6, 41, and 17% of our samples, respectively. Overall, 69 (69%) of the 100 samples were HPV positive; among these, 34/100 (34%) were positive for single HPV subtypes, while 35/100 (35%) of the samples were positive for two or more HPV subtypes. No significant association was noted between the presence of HPV and tumor grade, stage, or location. However, the presence of coinfection of HPV subtypes strongly correlated with advanced stage (stage 3 and 4) colorectal cancer, indicating that the copresence of more than one HPV subtype can significantly worsen the prognosis of colorectal cancer. The results from this study imply that coinfection with high-risk HPV subtypes is associated with the development of colorectal cancer in the Qatari population.

## 1. Introduction

Colorectal cancer (CRC) accounts for 10% of all cancer-related deaths worldwide and is the second most common cause of cancer deaths worldwide [1]. Moreover, CRC forms 13% of all malignant tumors in the gastrointestinal tract [2]. In addition, CRC represents the third most common type of cancer in males and the second in females [3]. Major risk factors associated with CRC are usually genetic, hereditary, or environmental, such as aging, lifestyle, and consumption of alcohol. Recent studies have linked the development of CRC with pathogenic viruses known as “oncoviruses” [4]. In this regard, oncoviruses can exploit or cooperate with other factors, such as compromised host-immune systems, chronic inflammatory conditions, or other carcinogens in order to initiate oncogenesis or sustain tumorigenesis [5,6]. The most commonly known oncoviruses include human papillomaviruses (HPVs), human herpes virus 8 (HHV8), Epstein–Barr virus (EBV), and hepatitis viruses B and C (HBV and HCV) [7].

HPVs are small, double-stranded DNA viruses that have the ability to infect the epithelial linings of the anogenital and upper respiratory tracts [8]. Numerous HPV types have been identified and classified into low-risk (LR) and high-risk (HR) groups based on their oncogenic ability. LR HPVs are generally involved in the development of common anogenital warts [9]. Additionally, infections induced by LR HPVs (HPV types −6 and −11) include benign papillomas/warts and respiratory papillomatosis [10,11]. Conversely, HR HPVs (16, 18, 31, 33, 35, 39, 45, 51, 52, 55, 56, 58, 59, 68, 73, 82, and 83) correlate with the onset and development of human cancers [12], as in cervical, head and neck, colorectal, breast, and certain subsets of genital cancers [8,10,13,14,15,16]. Moreover, persistent infection with HR HPVs was shown to correlate with vascular invasion, lymph node metastasis, as well as tumor grade and size [17,18,19,20].

The HPV genome is approximately 8 kb in size [21] and comprises an early region, late region, and a non-coding region (long control region: LCR) [21]. The early oncoprotein E5 deregulates cell survival and proliferation through EGF-R1-associated signaling pathways [22,23,24]. On the other hand, the early oncoproteins E6/E7 trigger the inhibition of apoptosis through the inactivation of known tumor suppressor proteins p53 and pRb [25]. In addition, E6/E7 cooperates with the ErbB-2 receptor to bring about D-type cyclins-mediated transformation in HPV infected cells [26,27,28], which occurs via the β-catenin singling pathway [29,30,31]. Moreover, E6/E7 can also induce the expression of biomarkers implicated in epithelial-to-mesenchymal transition (EMT) such as P-cadherin, Id-1, and Fascin, thus promoting increased cell proliferation, invasion, and metastasis [26,28,32,33,34,35]. Most HR HPVs are masters of immune escape, enabling persistent infections that can lead to complete neoplastic cellular transformation [8].

Several reports have highlighted that HPV infection is one of the potential causes of anal/rectal lesions that eventually lead to the development of CRC [36,37]. Sexually transmitted viruses such as HPV can initiate the development of warts and high-grade lesions in the epithelial lining of the anal canal and perianal skin that eventually progress to neoplasms of the anal and rectal regions [38,39]. HPV-associated anal and rectal cancers are most commonly linked to sexual activity involving HPV-infected participants [38,40,41]. However, persistent infection with HV is considered essential for implications of HPV-mediated cancers [42,43], thereby increasing the risk of developing CRC.

Nevertheless, only a few studies from the Middle East and North Africa (MENA) region have reported data on HPV-associated CRC. Moreover, investigations detailing a possible link between the presence of HPV and the onset of CRC among countries of the Gulf Cooperation Council (GCC) are scarce. Currently, only two studies from Saudi Arabia have evaluated the association of HPV in CRC [44,45]. Hence, this study will be the first from the State of Qatar to report the presence and copresence of different types of HR HPVs and their probable association with tumor stage and grade in CRC samples from Qatar.

## 2. Materials and Methods

### 2.1. Sample Collection and DNA Extraction

For the study, 100 formalin-fixed paraffin-embedded (FFPE) tissue samples from patients diagnosed during the period 2018–2021, at the Department of Pathology, Hamad Medical Corporation (HMC), were included. For the study, anonymization for all cases was performed. All experimental procedures were approved by the local research committee #IBC-2019/005.

All cases were re-reviewed by a board-certified pathologist to confirm the diagnosis and to select appropriate FFPE tissues for the assays.

DNA extraction from FFPE tissue samples (punch samples of 2 mm thickness) was performed using the Thermo Scientific GeneJET FFPE DNA Purification Kit according to the manufacturer’s instructions (ThermoFisher Scientific, Waltham, MA, USA). The enzymatic digestion of the FFPE sections was followed by lysing and release of genomic DNA. Afterwards, the DNA was de-crosslinked, and the solution was centrifuged to obtain the supernatant containing DNA, and binding was performed. Then, the lysate was added to the purification column, followed by washing adsorbed DNA to eradicate contaminants. Finally, DNA was eluted using the elution buffer (60 µL).

### 2.2. HPV Detection by PCR

Polymerase chain reaction (PCR) was performed to analyze HR HPVs in purified genomic DNA, through the use of primers specific to HR HPV types: 16, 18, 31, 33, 35, 45, 51, 52, and 59, as previously described [46,47]. For the internal control, GAPDH was used. All analyses were completed as described by our group [46]. For each single experiment, we used the respective positive and negative controls reported by our group previously [47].

PCR was performed using the Invitrogen Platinum II Hot-Start Green PCR Master Mix (2X) (ThermoFisher Scientific, Waltham, MA, USA) according to the manufacturer’s protocol. Gel electrophoresis was performed to resolve the PCR product, and images were captured using the iBrightCL1000 Imaging System (ThermoFisher Scientific, Waltham, MA, USA).

### 2.3. Statistical Analysis

Chi-square (χ2) test with Yates’ correction and Fisher’s exact test were performed to determine the significant association between the presence and copresence of high-risk HPVs with clinicopathological data (tumor grade, tumor stage, and lymph node involvement). Statistical significance was achieved if *p*-values were ≤0.05 in two-tailed tests. Logistic regression was performed to estimate the association of HPV and its subtypes with clinical correlates. HPV infection was coded as 1 and 0, indicating presence and absence of infection. Next, subtypes of HPV were combined to create a binary variable coinfection indicating more than two subtypes (2 or more) as present, and less as absent. Stage of Cancer was classified as an advanced binary variable (stages 3 and 4) and others (stages 1 and 2). Statistical analysis and plotting of graphs were performed using the Stata software (version 17).

## 3. Results

### 3.1. Clinicopathological Characteristics of the Cohort

The study included 100 samples from 66 (66.0%) male and 34 (34.0%) female CRC patients from Qatar, with a mean age of 57.1 years and a standard deviation of ±13.9 years. Most samples were taken from the sigmoid colon (23 cases, 23%), followed by the rectosigmoid colon (17 cases, 17%), ascending colon (16 cases, 16%), and the descending colon (14 cases, 14%), while the rest of the samples were obtained from other varying regions of the colon (30 cases, 30%), as shown in Table 1.

All tumors were histologically confirmed as adenocarcinomas, with most being grade 2 (84%). Based on the pT stage, 11 (11%) of the cases were stage 1, 28 (28%) cases were stage 2, 45 (45%) cases were stage 3, and 16 (16%) cases were identified to have pT4 stage disease during sample collection. A total of 58 cases (58%) were found to have lymph node metastases. (Table 1).

### 3.2. The Status of High-Risk HPVs and Their Association with Clinicopathological Characteristics

**Figure 1:**
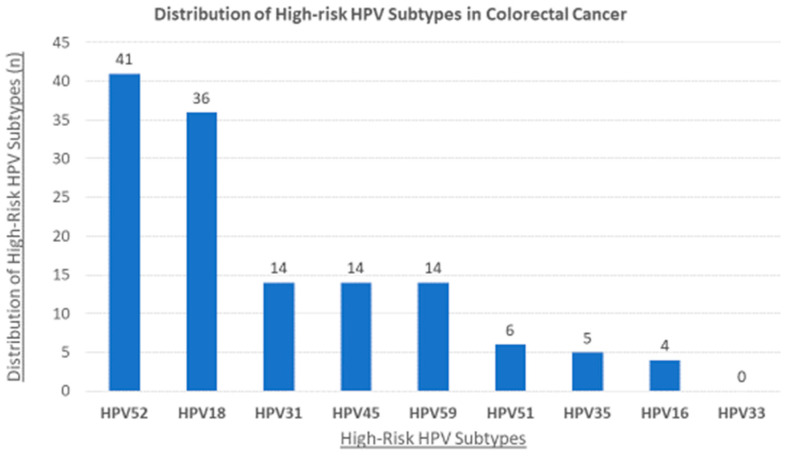
The distribution of high-risk HPV subtypes in Qatari colorectal samples. The PCR analysis included 100 colorectal samples revealing that the most frequent human papillomavirus (HPV) subtypes in Qatar are 52, 18, 31, 45, 59, 51, 35, and 16.

In this investigation, we examined the presence of HR HPV types in a cohort of 100 CRC samples from the Qatari population by PCR analysis using specific primers for their E6/E7 genes. Our study revealed that 69 of the 100 samples in our cohort were HPV positive (69%); the most commonly present HR HPVs were HPV52 (41%), followed by HPV18 (36%), HPV59 (17%), HPVs 31 and 45 (14%, each), HPV51 (6%), HPV35 (5%), and HPV16 (4%) (Figure 1). However, HPV type 33 was not detected in our examined samples.

Out of the 66 male cases, 41 (59%) were HPV positive, whereas, among the 34 female cases, 28 (41%) were positive for HPVs. Univariate logistic regression analysis revealed an odds ratio of 2.84, signifying that the odds of females with HPV-associated CRC are nearly three times greater than those of men (*p* = 0.043). However, the presence of HPV did not correlate with the anatomical site of the tumor and the number of lymph nodes affected. Table 1 summarizes the clinicopathological characteristics of the HPV-positive and negative cases.

Furthermore, our data revealed that 34/100 (34%) cases were positive for only one HPV subtype, while 35/100 (35%) were found to be co-infected with more than one type of HPV. Table 2 summarizes the types and frequencies of HPV coinfections in the CRC cohort. A total of 16/100 (16%) cases were found to have double infections, with HPV18 and HPV52 being the most commonly observed coinfections (n = 4) within this group. Additionally, 8/100 cases showed the co-presence of three HPV subtypes with HPV18, HPV52, and HPV59 being the most frequent combination (n = 4). In the quadruple-infections group (8/100 (8%)), HPV18, HPV31, HPV45, and HPV52 was the most frequently observed combination of HPV subtypes (n = 3). Lastly, only 3/100 cases were found to possess quintuple infections, with two of them being infected with HPV18, HPV31, HPV45, HPV51, and HPV52.

The coinfection of HPV subtypes was found to strongly correlate with advanced stage (stage 3 and 4) CRC, indicating that the copresence of more than one HPV subtype can significantly worsen the prognosis of colorectal cancer. On assessing overall HPV infection status, the odds of having colorectal cancer are 1.58 times higher in those infected with HPV than those who do not have an infection. The results indicate that a 95% confidence interval of (0.64–4.11) is wide and (*p*-value of 0.305) is not statistically significant, which could be due to the small cohort size (Table 3). However, the results changed when the same association was examined with coinfection of HPV subtypes. Our results indicate that the odds of colorectal cancer occurring in the group infected with two or more subtypes of HPV are 4.10 times higher than in the group with no or single-subtype HPV infection. The 95% confidence interval of (1.57–11.68) indicates a 95% probability that the true odds ratio falls between 1.57 and 11.68, with a *p*-value of 0.004 (Table 4). Thus, the presence of coinfection of HR HPVs may be considered as a high-risk factor for the progression of CRC.

## 4. Discussion

### 4.1. Detection of HPV among CRC Samples from a Qatari Population

As evidenced by the existing scientific literature, and to the best of our knowledge, this study is the first of its kind to reveal the presence and copresence of high-risk HPV types in 100 CRC samples from the State of Qatar.

According to our study, 69 of the 100 samples (69%) were found to be positive for the presence of high-risk HPV types. Unfortunately, data on the presence of high-risk HPVs arising from countries in the GCC as well as the MENA region are scarce, as only a few countries have reported the presence of HPV in CRC. However, there appears to be a lack of consensus on HPV prevalence among these countries; some countries are known to report low HPV positivity, while others report relatively higher numbers. Among the countries in the GCC region, studies from the Kingdom of Saudi Arabia (KSA) are known to report a prevalence of HPV in CRC. Among them, one study reported a low prevalence of HPV (1.5%) [44], while another by Khabaz [45] reported the complete absence of HPV in CRC samples. In contrast to these studies, our study from Qatar reports a relatively high prevalence (69%) of HPV in CRC. Moreover, since our study reports a fairly low prevalence of HPV 16 (4%) infections in the Qatari CRC population, the absence of HPV reported by Khabaz [45] may be due to the use of detection methods that were based on detecting only HPV 16 and HPV 18 infections, which may be low-occurring in the GCC region. Further, in the larger MENA region, a higher prevalence of HPV in CRC was noted in Syria (54%) [32] and Lebanon (64%) [50], which is comparable to the results from our study. Interestingly, the highest prevalence of HPV in CRC in the MENA region comes from Turkey, where studies reported 81% [51] and 82% [52] of CRC samples to have HPV infections.

Moreover, although reports on HPVs association with CRC are rare in MENA countries, a bulk of the currently available data from this region arises from Iran. However, it is indeed surprising to note that only one out of the nine studies from Iran reported a high prevalence of HPVs (83%) [53]. The remaining majority of the studies report a relatively lower prevalence within the range of 0% to 23% [54,55,56,57,58,59,60,61]. Similarly, most other studies from the MENA region report a lower prevalence of HPV in CRC. For example, two studies from Egypt found HPVs association in only 15% [62] and 22% [63] of CRC samples tested. In addition, one report from Iraq revealed 44% HPV positivity in CRC [64]. Data from these studies do not align with the higher presence of high-risk HPVs (69%), as noted in our study. Such discrepancies could be attributed to the varying sizes of these studies, the population diversity, and the technique sensitivity used to detect high-risk HPV types. Most importantly, although HPVs 16 and 18 are the most frequently observed high-risk HPVs globally and were the focus of studies conducted in the GCC and MENA regions, nevertheless, based on our findings on HPV genotyping in this region, they do not appear to be the most prevalent types [32,50,65]. Globally, numerous reports from France [66], Brazil [67], and Cuba [40] have shown HPV types 16 and 18 to be the most oncogenic HPVs associated with the pathogenesis of CRC. However, the frequent observation of these two HPVs in Western countries cannot be extrapolated to other ethnic populations.

This study identifies the most commonly occurring HPV types in Qatari CRC samples to be HPVs 52, 18, 59, 31, 45, 51, 35, and 16 in descending order. Accordingly, a study using breast cancer samples revealed that high-risk HPV type 52 is among the most frequent in the Qatari population [68]. In accordance with our data, other reports arising from nations within the MENA region identified HPVs 16, 18, 51, and 58 as the most prevalent high-risk HPV subtypes in CRC [32,50,56,57,69,70]. Moreover, data from our study are similar to other reports from Lebanon, Turkey, Iran, Syria, and Israel, where HPVs 16, 18, 31, and 35 were the highest reported high-risk HPV subtypes [32,50,54,56,57,58,65]. Contrary to findings from Turkey [51,52,71] and Syria [32] in CRC, we did not observe the presence of HPV33 in any of our samples.

These findings strongly suggest that HPV detection in the MENA region including GCC countries should not be based solely on screening for HPV16 and HPV18, as the prevalence of high-risk HPV genotypes can potentially vary from one region to another, as mentioned above.

### 4.2. Correlation between the Presence of HPV and Clinicopathological Characteristics of the CRC Patients

Our study aimed to evaluate the presence/copresence of HR HPV subtypes and their association with CRC phenotype. We noted that the presence of HPV did not correlate with specific clinicopathological characteristics of the CRC cohort, namely the anatomical site of the tumor, number of lymph nodes affected, tumor grade/stage, and metastasis (Table 1). While, it is important to highlight that several studies reported E6 of HPV type 16 to bind and inactivate p53 and pRb expression [25,72,73,74]. An investigation by Chen et al. [74] in E6-positive/p53-mutated tumors reported loss of p21 and mdm2 mRNA expression levels via p53 inhibition, indicating a plausible role of HPV in CRC pathogenesis. Additionally, E6 induces telomerase activation, altering pathways regulating cellular proliferation, differentiation, immune recognition, and survival signaling [25]. Accordingly, our group pointed out the ability of E6/E7 of HPV type 16 to transform human normal mesenchymal colorectal cells, and promote the migratory capacity of transformed cells. In addition to this study, we demonstrated the ability of E6/E7 of HPV type 16 to convert non-invasive and non-metastatic cancer cells into invasive and metastatic ones, respectively [26]. These data indicate that HR HPVs alone could contribute towards playing a crucial role in the onset and progression of human colorectal carcinomas.

### 4.3. Copresence of HR-HPV Subtypes in CRC

According to our data, out of the 69 cases that were positive for HPV, 34/69 (49.2%) were found to have single infections, with the rest 35/69 (50.8%) being co-infected with more than one HPV subtype. These figures indicate that more than half of positive HPV cases are co-infected with two, three, four, or five HPV subtypes. These results are in contrast with those reported by other studies [17,75,76,77], where single HPV infections are more common than multiple-HPV in cancers. Such a high occurrence of coinfections in this population is indeed noteworthy.

In addition, we found that coinfection with different HR-HPV subtypes strongly correlates with advanced stage (stage 3 and 4) CRC. This is a strong indicator that the copresence of HR HPV subtypes significantly worsens the prognosis of CRC. In the present study, we show a significant association between coinfection by high-risk subtypes and CRC, with odds 4.10 times higher in the group infected with two or more HPV types, as compared to the groups without HPV or with single HPV infections. These findings are in line with most other studies which also report similar associations between multiple HPV infections and disease severity, and/or decline in treatment response in cancer patients [78,79,80]. Such inferences have important implications for understanding the role of HR HPVs in colorectal carcinogenesis, which highlights the need for further research to investigate the mechanisms underlying this association, as well as the potential for HPV-based prevention and therapeutic strategies for colorectal cancer.

## 5. Conclusions

We conclude that high-risk HPV types 16, 18, 31, 35, 45, 51, 52, and 59 are present in human CRC in the Qatari population. In addition, HPV types 18 and 52 are the most dominant types of HPV infection in Qatar. Among the infected cases, the presence of coinfection with more than one HPV subtype is particularly high (50.8%) in our cohort. More importantly, our findings point out that HR HPVs coinfection in human CRC can play an important role in its progression, since it is clearly associated with advanced tumor stage. Therefore, HPV vaccines must be utilized in order to prevent CRC initiation or progression [81]; in this regard, most of the currently available vaccines for HPV are based on offering protection against HPVs 16 and 18 as the main two HR HPV types [82,83,84]. Therefore, the recent 9-valent HPV vaccine [85] is maybe the best suited vaccine based on our study. Nevertheless, we believe that further investigation with a larger cohort from the Gulf region is necessary in order to validate our findings.

## Figures and Tables

**Table 1:** Summarizing the clinicopathological characteristics of the CRC cohort and its association with the presence of HPV. The mean age of all patients is 57.1 years (standard deviation (SD), ±13.9 years).

Characteristic	Category	HPV^−^ (n = 31)	HPV^+^ (n = 69)	*p*-Value
Age in years (median)		59.0 (47.0, 65.0)	60.0 (49.0, 67.0)	0.61
Gender	Male	25 (81%)	41 (59%)	0.038
	Female	6 (19%)	28 (41%)	
Tumor Location	Ascending colon	5 (16%)	11 (16%)	0.83
	Descending colon	4 (13%)	10 (14%)	
	Transverse colon	2 (6%)	3 (4%)	
	Sigmoid colon	5 (16%)	18 (26%)	
	Rectosigmoid	7 (23%)	10 (14%)	
	Cecum	4 (13%)	6 (9%)	
	Rectum	1 (3%)	2 (3%)	
	Hepatic flexure	3 (10%)	4 (6%)	
	Splenic flexure	0 (0%)	2 (3%)	
	Others (ileocecal valve, appendix)	0 (0%)	3 (4%)	
Tumor Grade *	Not Applicable	0 (0%)	1 (1%)	0.41
	Grade 1 (Well Differentiated)	2 (6%)	3 (4%)	
	Grade 2 (Moderately Differentiated)	28 (90%)	56 (81%)	
	Grade 3 (Poorly Differentiated)	1 (3%)	9 (13%)	
Tumor Stage ^☥^	pT1	0 (0%)	1 (1%)	0.45
	pT2	6 (19%)	7 (10%)	
	pT3	19 (61%)	51 (74%)	
	pT4	6 (19%)	10 (14%)	
Lymph Node Involvement (pN)	pN0	15 (48%)	27 (39%)	0.69
	pN1	10 (32%)	26 (38%)	
	pN2	6 (19%)	16 (23%)	
Metastasis	Absent	28 (90%)	56 (81%)	0.25
	Present	3 (10%)	13 (19%)	

HPV^−^ denotes the cases negative for HPV; HPV^+^ denotes the cases positive for HPV; * Tumor grade is determined according to the College of American Pathologists Consensus Statement [48]. ^☥^ Tumor stage is based on the American Joint Committee on Cancer (AJCC) TNM system (8th edition) [49].

**Table 2:** Classifying the types of HPV coinfection and most frequently occurring combinations among our CRC cohort from Qatar.

Coinfection of HR-HPV Subtypes (n = 35)	
HPV Subtypes	Cases
Double-infections (n = 16) 16%	
HPV18 and HPV52	4
HPV52 and HPV45	3
HPV52 and HPV35	2
HPV18 and HPV59	2
HPV52 and HPV59	2
HPV18 and HPV31	2
HPV16 and HPV18	1
Triple-infections (n = 8) 8%	
HPV18, HPV52, and HPV59	4
HPV18, HPV35, and 52	1
HPV18, HPV45, and HPV52	1
HPV18, HPV31, and HPV52	1
HPV45, HPV51, and HPV52	1
Quadruple-infections (n = 8) 8%	
HPV18, HPV31, HPV45, and HPV52	3
HPV18, HPV45, HPV52, and HPV59	1
HPV18, HPV31, HPV35, and HPV52	1
HPV16, HPV18, HPV45, and HPV52	1
HPV35, HPV45, HPV52, and HPV59	1
HPV18, HPV51, HPV52, and HPV59	1
Quintuple-infections (n = 3) 3%	
HPV18, HPV31, HPV45, HPV51, and HPV52	2
HPV18, HPV31, HPV45, HPV52, and HPV59	1

**Table 3:** Association between advanced tumor stage * and HPV infection (n = 100).

Variables		Odds Ratio	95% Confidence Interval	*p* Value
Unadjusted	HPV	1.44	0.61–3.42	0.399
Adjusted		1.62	0.64–4.11	0.305
	Age	0.95	0.92–0.98	0.009
	Sex	0.85	0.34–2.12	0.731
	Location	0.99	0.83–1.19	0.989

* Advanced = stages 3 and 4; HPV infection is infection with any subtype.

**Table 4:** Association between advanced tumor stage * and HPV Coinfection ^#^. (n = 100).

Variables		Odds Ratio	95% Confidence Interval	*p* Value
Unadjusted	HPV coinfection	3.87	1.48–10.13	0.0033
Adjusted		4.28	1.57–11.68	0.004
	Age	0.95	0.92–0.98	0.009
	Sex	0.850	0.31–2.105	0.647
	Location	0.998	0.81–1.19	0.856

* Advanced = stage 3 and 4. ^#^ HPV infection is infection with two or more subtypes. Adjusted for Age, Gender, and Site of Colorectal cancer.

## Data Availability

The data presented in this study is contained within the article.

## References

[1] Sung H., Ferlay J., Siegel R.L., Laversanne M., Soerjomataram I., Jemal A., Bray F. (2021). Global Cancer Statistics 2020: GLOBOCAN Estimates of Incidence and Mortality Worldwide for 36 Cancers in 185 Countries. CA Cancer J. Clin..

[2] Kim H.S., Heo J.S., Lee J., Lee J.Y., Lee M.-Y., Lim S.H., Lee W.Y., Kim S.H., Park Y.A., Cho Y.B. (2016). The impact of KRAS mutations on prognosis in surgically resected colorectal cancer patients with liver and lung metastases: A retrospective analysis. BMC Cancer.

[3] Hossain M.S., Karuniawati H., Jairoun A.A., Urbi Z., Ooi J., John A., Lim Y.C., Kibria K.M.K., Mohiuddin A.K.M., Ming L.C. (2022). Colorectal Cancer: A Review of Carcinogenesis, Global Epidemiology, Current Challenges, Risk Factors, Preventive and Treatment Strategies. Cancers.

[4] Marongiu L., Allgayer H. (2022). Viruses in colorectal cancer. Mol. Oncol..

[5] zur Hausen H. (2009). The search for infectious causes of human cancers: Where and why. Virology.

[6] Bouvard V., Baan R., Straif K., Grosse Y., Secretan B., El Ghissassi F., Benbrahim-Tallaa L., Guha N., Freeman C., Galichet L. (2009). A review of human carcinogens—Part B: Biological agents. Lancet Oncol..

[7] White M.K., Pagano J.S., Khalili K. (2014). Viruses and human cancers: A long road of discovery of molecular paradigms. Clin. Microbiol. Rev..

[8] Tommasino M. (2014). The human papillomavirus family and its role in carcinogenesis. Semin. Cancer Biol..

[9] Stanley M.A. (2012). Genital human papillomavirus infections: Current and prospective therapies. J. Gen. Virol..

[10] Lacey C.J.N., Lowndes C.M., Shah K.V. (2006). Chapter 4: Burden and management of non-cancerous HPV-related conditions: HPV-6/11 disease. Vaccine.

[11] Jamshidi M., Shekari M., Nejatizadeh A.A., Malekzadeh K., Baghershiroodi M., Davudian P., Dehghan F., Jamshidi F. (2012). The impact of human papillomavirus (HPV) types 6, 11 in women with genital warts. Arch. Gynecol. Obstet..

[12] Bernard H.U., Burk R.D., Chen Z., van Doorslaer K., zur Hausen H., de Villiers E.M. (2010). Classification of papillomaviruses (PVs) based on 189 PV types and proposal of taxonomic amendments. Virology.

[13] Chan C.K., Aimagambetova G., Ukybassova T., Kongrtay K., Azizan A. (2019). Human Papillomavirus Infection and Cervical Cancer: Epidemiology, Screening, and Vaccination—Review of Current Perspectives. J. Oncol..

[14] Fernandes Q., Gupta I., Vranic S., Al Moustafa A.E. (2020). Human papillomaviruses and epstein–barr virus interactions in colorectal cancer: A brief review. Pathogens.

[15] Syrjänen K.J. (2002). HPV infections and oesophageal cancer. J. Clin. Pathol..

[16] Delgado-García S., Martínez-Escoriza J.-C., Alba A., Martín-Bayón T.-A., Ballester-Galiana H., Peiró G., Caballero P., Ponce-Lorenzo J. (2017). Presence of human papillomavirus DNA in breast cancer: A Spanish case-control study. BMC Cancer.

[17] Ohadian Moghadam S., Mansori K., Nowroozi M.R., Afshar D., Abbasi B., Nowroozi A. (2020). Association of human papilloma virus (HPV) infection with oncological outcomes in urothelial bladder cancer. Infect. Agent. Cancer.

[18] Graflund M., Sorbe B., Sigurdardóttir S., Karlsson M. (2004). HPV-DNA, vascular space invasion, and their impact on the clinical outcome in early-stage cervical carcinomas. Int. J. Gynecol. Cancer.

[19] Lei J., Ploner A., Elfström K.M., Wang J., Roth A., Fang F., Sundström K., Dillner J., Sparén P. (2020). HPV Vaccination and the Risk of Invasive Cervical Cancer. N. Engl. J. Med..

[20] Lukaszuk K., Liss J., Wozniak I., Sliwinski W., Emerich J., Wojcikowski C. (2004). HPV and histological status of pelvic lymph node metastases in cervical cancer: A prospective study. J. Clin. Pathol..

[21] Bernard H.-U., Calleja-Macias I.E., Dunn S.T. (2005). Genome variation of human papillomavirus types: Phylogenetic and medical implications. Int. J. Cancer.

[22] Kim S.-H., Juhnn Y.-S., Kang S., Park S.-W., Sung M.-W., Bang Y.-J., Song Y.-S. (2006). Human papillomavirus 16 E5 up-regulates the expression of vascular endothelial growth factor through the activation of epidermal growth factor receptor, MEK/ ERK1,2 and PI3K/Akt. Cell. Mol. Life Sci..

[23] Suprynowicz F.A., Disbrow G.L., Krawczyk E., Simic V., Lantzky K., Schlegel R. (2007). HPV-16 E5 oncoprotein upregulates lipid raft components caveolin-1 and ganglioside GM1 at the plasma membrane of cervical cells. Oncogene.

[24] Oh J.-M., Kim S.-H., Cho E.-A., Song Y.-S., Kim W.-H., Juhnn Y.-S. (2009). Human papillomavirus type 16 E5 protein inhibits hydrogen peroxide-induced apoptosis by stimulating ubiquitin-proteasome-mediated degradation of Bax in human cervical cancer cells. Carcinogenesis.

[25] Tomaić V. (2016). Functional roles of E6 and E7 oncoproteins in HPV-induced malignancies at diverse anatomical sites. Cancers.

[26] Yasmeen A., Bismar T.A., Kandouz M., Foulkes W.D., Desprez P.-Y., Al Moustafa A.-E. (2007). E6/E7 of HPV Type 16 Promotes Cell Invasion and Metastasis of Human Breast Cancer Cells. Cell Cycle.

[27] Yasmeen A., Hosein A.N., Yu Q., Al Moustafa A.-E. (2007). Critical role for D-type cyclins in cellular transformation induced by E6/E7 of human papillomavirus type 16 and E6/E7/ErbB-2 cooperation. Cancer Sci..

[28] Al Moustafa A.-E., Foulkes W.D., Wong A., Jallal H., Batist G., Yu Q., Herlyn M., Sicinski P., Alaoui-Jamali M.A. (2004). Cyclin D1 is essential for neoplastic transformation induced by both E6/E7 and E6/E7/ErbB-2 cooperation in normal cells. Oncogene.

[29] Yasmeen A., Alachkar A., Dekhil H., Gambacorti-Passerini C., Al Moustafa A.-E. (2010). Locking Src/Abl Tyrosine Kinase Activities Regulate Cell Differentiation and Invasion of Human Cervical Cancer Cells Expressing E6/E7 Oncoproteins of High-Risk HPV. J. Oncol..

[30] Coluccia A.M.L., Benati D., Dekhil H., De Filippo A., Lan C., Gambacorti-Passerini C. (2006). SKI-606 decreases growth and motility of colorectal cancer cells by preventing pp60(c-Src)-dependent tyrosine phosphorylation of beta-catenin and its nuclear signaling. Cancer Res..

[31] Al Moustafa A.-E., Kassab A., Darnel A., Yasmeen A. (2008). High-Risk HPV/ErbB-2 Interaction on E-Cadherin/Catenin Regulation in Human Carcinogenesis. Curr. Pharm. Des..

[32] Al Moustafa A.-E., Ghabreau L., Segal E., Yasmeen A., Kassab A., Akil N. (2012). High-risk human papillomavirus infections in colorectal cancer in the Syrian population and their association with Fascin, Id-1 and P-cadherin expressions: A tissue microarray study. Clin. Cancer Investig. J..

[33] Oh S.Y., Kim Y.B., Suh K.W., Paek O.J., Moon H.Y. (2012). Prognostic Impact of Fascin-1 Expression is More Significant in Advanced Colorectal Cancer. J. Surg. Res..

[34] Ling M.-T., Wang X., Zhang X., Wong Y.-C. (2006). The multiple roles of Id-1 in cancer progression. Differentiation.

[35] Van Marck V., Stove C., Jacobs K., Van den Eynden G., Bracke M. (2010). P-cadherin in adhesion and invasion: Opposite roles in colon and bladder carcinoma. Int. J. Cancer.

[36] Wang C.-C.J., Sparano J., Palefsky J.M. (2017). Human Immunodeficiency Virus/AIDS, Human Papillomavirus, and Anal Cancer. Surg. Oncol. Clin. N. Am..

[37] Assi R., Reddy V., Einarsdottir H., Longo W.E. (2014). Anorectal human papillomavirus: Current concepts. Yale J. Biol. Med..

[38] Frisch M., Glimelius B., van den Brule A.J.C., Wohlfahrt J., Meijer C.J.L.M., Walboomers J.M.M., Goldman S., Svensson C., Adami H.-O., Melbye M. (1997). Sexually Transmitted Infection as a Cause of Anal Cancer. N. Engl. J. Med..

[39] Beckmann A.M., Daling J.R., Sherman K.J., Maden C., Miller B.A., Coates R.J., Kiviat N.B., Myerson D., Weiss N.S., Hislop T.G. (1989). Human papillomavirus infection and anal cancer. Int. J. Cancer.

[40] Limia C.M., Soto Y., García Y., Blanco O., Kourí V., López M.V., Toledo M.E., Pérez L., Baños Y., Caturla Y. (2017). Human papillomavirus infection in anal intraepithelial lesions from HIV infected Cuban men. Infect. Agent. Cancer.

[41] Moscicki A.B., Darragh T.M., Michael Berry-Lawhorn J., Roberts J.M., Khan M.J., Boardman L.A., Chiao E., Einstein M.H., Goldstone S.E., Jay N. (2015). Screening for anal cancer in women. J. Low. Genit. Tract Dis..

[42] Einstein M.H., Burk R.D. (2001). Persistent human papillomavirus infection: Definitions and clinical implications. Papillomavirus Rep..

[43] Schiffman M., Wentzensen N. (2010). From human papillomavirus to cervical cancer. Obstet. Gynecol..

[44] Gazzaz F., Mosli M.H., Jawa H., Sibiany A. (2016). Detection of human papillomavirus infection by molecular tests and its relation to colonic polyps and colorectal cancer. Saudi Med. J..

[45] Khabaz M.N. (2016). HPV and the Development of Colorectal Cancer. Glob. J. Health Sci..

[46] Gupta I., Al Farsi H., Jabeen A., Skenderi F., Al-Thawadi H., AlAhmad Y.M., Al Moustafa A.-E., Vranic S. (2020). High-Risk Human Papillomaviruses and Epstein-Barr Virus in Colorectal Cancer and Their Association with Clinicopathological Status. Pathogens.

[47] Gupta I., Ulamec M., Peric-Balja M., Ramic S., Al Moustafa A.-E., Vranic S., Al-Farsi H.F. (2021). Presence of high-risk HPVs, EBV, and MMTV in human triple-negative breast cancer. Hum. Vaccin. Immunother..

[48] Compton C.C., Fielding L.P., Burgart L.J., Conley B., Cooper H.S., Hamilton S.R., Hammond M.E.H., Henson D.E., Hutter R.V.P., Nagle R.B. (2000). Prognostic factors in colorectal cancer: College of American Pathologists consensus statement 1999. Arch. Pathol. Lab. Med..

[49] Weiser M.R. (2018). AJCC 8th Edition: Colorectal Cancer. Ann. Surg. Oncol..

[50] Nagi K., Gupta I., Jurdi N., Yasmeen A., Vranic S., Batist G., Al Moustafa A.-E. (2021). Copresence of High-Risk Human Papillomaviruses and Epstein-Barr Virus in Colorectal Cancer: A Tissue Microarray and Molecular Study from Lebanon. Int. J. Mol. Sci..

[51] Buyru N., Tezol A., Dalay N. (2006). Coexistence of K-ras mutations and HPV infection in colon cancer. BMC Cancer.

[52] Salepci T., Yazici H., Dane F., Topuz E., Dalay N., Onat H., Aykan F., Seker M., Aydiner A. (2009). Detection of human papillomavirus DNA by polymerase chain reaction and southern blot hybridization in colorectal cancer patients. J. BUON.

[53] Damin D.C., Caetano M.B., Rosito M.A., Schwartsmann G., Damin A.S., Frazzon A.P., Ruppenthal R.D., Alexandre C.O.P. (2007). Evidence for an association of human papillomavirus infection and colorectal cancer. Eur. J. Surg. Oncol..

[54] Karbalaie Niya M.H., Keyvani H., Safarnezhad Tameshkel F., Salehi-Vaziri M., Teaghinezhad-S S., Bokharaei Salim F., Monavari S.H.R., Javanmard D. (2018). Human Papillomavirus Type 16 Integration Analysis by Real-time PCR Assay in Associated Cancers. Transl. Oncol..

[55] Karbasi A., Borhani N., Daliri K., Kazemi B., Manoochehri M. (2015). Downregulation of external death receptor genes FAS and DR5 in colorectal cancer samples positive for human papillomavirus infection. Pathol. Res. Pract..

[56] Mahmoudvand S., Safaei A., Erfani N., Sarvari J. (2015). Presence of Human Papillomavirus DNA in Colorectal Cancer Tissues in Shiraz, Southwest Iran. Asian Pac. J. Cancer Prev..

[57] Afshar R.M., Deldar Z., Mollaei H.R., Arabzadeh S.A., Iranpour M. (2018). Evaluation of HPV DNA positivity in colorectal cancer patients in Kerman, Southeast Iran. Asian Pac. J. Cancer Prev..

[58] Meshkat M., Tayyebi Meibodi N., Sepahi S., Fadaee N., Salehpour M., Meshkat Z. (2014). The frequency of human papillomaviruses in colorectal cancer samples in Mashhad, northeastern Iran. TURKISH J. Med. Sci..

[59] Ranjbar R., Saberfar E., Shamsaie A., Ghasemian E. (2014). The Aetiological Role of Human Papillomavirus in Colorectal Carcinoma: An Iranian Population- Based Case Control Study. Asian Pac. J. Cancer Prev..

[60] Taherian H., Tafvizi F., Fard Z.T., Abdirad A. (2014). Lack of association between human papillomavirus infection and colorectal cancer. Prz. Gastroenterol..

[61] Tavakolian S., Goudarzi H., Eslami G., Dayyani F., Kazeminezhad B., Faghihloo E. (2020). Prevalence of human papilloma virus and Epstein–Barr virus in tumorous and adjacent tissues of colorectal cancer in Iran. Gene Rep..

[62] El-Seidi E.A., Sorour A.E., Gamil M. (2014). Human Papillomavirus in Patients with Colorectal Cancer. Egypt J. Med. Microbiol..

[63] Hafez F.S., Meckawy G.R., Alorabi M., Shakweer M.M. (2022). Interpretation of P16 expression as a marker of HPV in colorectal cancer. Histol. Histopathol..

[64] Kadhem Mallakh M., Mohammed Mahmood M., Hasan Mohammed Ali S. (2022). Immunomolecular Investigation of Human Papillomavirus Genotypes (16, 18) and P63 Expression in Patients with Malignant and Non-malignant Colorectal Tumors. Arch. Razi Inst..

[65] Malki M.I., Gupta I., Fernandes Q., Aboulkassim T., Yasmeen A., Vranic S., Al Moustafa A.E., Al-Thawadi H.A. (2020). Co-presence of Epstein–Barr virus and high-risk human papillomaviruses in Syrian colorectal cancer samples. Hum. Vaccines Immunother..

[66] Alberts C.J., Heard I., Canestri A., Marchand L., Fléjou J.-F., Piroth L., Ferry T., Didelot J.-M., Siproudhis L., Henno S. (2019). Incidence and Clearance of Anal Human Papillomavirus (HPV)-16 and HPV-18 Infection, and Their Determinants, Among Human Immunodeficiency Virus-Infected Men Who Have Sex With Men in France. J. Infect. Dis..

[67] de Jesus S.P., da Costa A.C.M., Barcellos R.B., de Medeiros R.M., da Silva C.M.D., Rossetti M.L. (2018). A high prevalence of human papillomavirus 16 and 18 co-infections in cervical biopsies from southern Brazil. Braz. J. Microbiol..

[68] Gupta I., Jabeen A., Al-Sarraf R., Farghaly H., Vranic S., Sultan A.A., Al Moustafa A.-E., Al-Thawadi H. (2021). The co-presence of high-risk human papillomaviruses and Epstein-Barr virus is linked with tumor grade and stage in Qatari women with breast cancer. Hum. Vaccin. Immunother..

[69] Motlagh A., Azadeh P., Hashemi M., Shafaghi B., Nouri N.B., Moulaei M., Sheybani K.H.M., Fazl A.A., Foudazi M., Velaei N. (2007). Human Papillomavirus Infection, P53 over Expression and Histopathologic Characteristics in Colorectal Cancer. Govaresh.

[70] Tanzi E., Bianchi S., Frati E.R., Amicizia D., Martinelli M., Bragazzi N.L., Brisigotti M.P., Colzani D., Fasoli E., Zehender G. (2015). Human papillomavirus detection in paraffin-embedded colorectal cancer tissues. J. Gen. Virol..

[71] Sayhan N., Yazici H., Budak M., Bitisik O., Dalay N. (2001). P53 codon 72 genotypes in colon cancer. Association with human papillomavirus infection. Res. Commun. Mol. Pathol. Pharmacol..

[72] Riley R.R., Duensing S., Brake T., Münger K., Lambert P.F., Arbeit J.M. (2003). Dissection of human papillomavirus E6 and E7 function in transgenic mouse models of cervical carcinogenesis. Cancer Res..

[73] Hengstermann A., Linares L.K., Ciechanover A., Whitaker N.J., Scheffner M. (2001). Complete switch from Mdm2 to human papillomavirus E6-mediated degradation of p53 in cervical cancer cells. Proc. Natl. Acad. Sci. USA.

[74] Chen T.-H., Huang C.-C., Yeh K.-T., Chang S.-H., Chang S.-W., Sung W.-W., Cheng Y.-W., Lee H. (2012). Human papilloma virus 16 E6 oncoprotein associated with p53 inactivation in colorectal cancer. World J. Gastroenterol..

[75] Correa R.M., Baena A., Valls J., Colucci M.C., Mendoza L., Rol M., Wiesner C., Ferrera A., Fellner M.D., González J.V. (2022). Distribution of human papillomavirus genotypes by severity of cervical lesions in HPV screened positive women from the ESTAMPA study in Latin America. PLoS ONE.

[76] Mbulawa Z.Z.A., Phohlo K., Garcia-Jardon M., Williamson A.L., Businge C.B. (2022). High human papillomavirus (HPV)-35 prevalence among South African women with cervical intraepithelial neoplasia warrants attention. PLoS ONE.

[77] Mpunga T., Baena A., Valls J., Colucci M.C., Mendoza L., Rol M., Wiesner C., Ferrera A., Fellner M.D., González J.V. (2020). Human papillomavirus genotypes in cervical and other HPV-related anogenital cancer in Rwanda, according to HIV status. Int. J. Cancer.

[78] Badawi H., Ahmed H., Ismail A., Diab M., Moubarak M., Badawy A., Saber M. (2008). Role of human papillomavirus types 16, 18, and 52 in recurrent cystitis and urinary bladder cancer among Egyptian patients. Medscape J. Med..

[79] Kaliff M., Sorbe B., Mordhorst L.B., Helenius G., Karlsson M.G., Lillsunde-Larsson G. (2018). Findings of multiple HPV genotypes in cervical carcinoma are associated with poor cancer-specific survival in a Swedish cohort of cervical cancer primarily treated with radiotherapy. Oncotarget.

[80] Munagala R., Doná M.G., Rai S.N., Jenson A.B., Bala N., Ghim S.J., Gupta R.C. (2009). Significance of multiple HPV infection in cervical cancer patients and its impact on treatment response. Int. J. Oncol..

[81] Fernandes Q., Allouch S., Gupta I., Elmakaty I., Elzawawi K.E., Amarah A., Al-Thawadi H., Al-Farsi H., Vranic S., Al Moustafa A.-E. (2022). Human Papillomaviruses-Related Cancers: An Update on the Presence and Prevention Strategies in the Middle East and North African Regions. Pathogens.

[82] Cheng L., Wang Y., Du J. (2020). Human papillomavirus vaccines: An updated review. Vaccines.

[83] Harper D.M., DeMars L.R. (2017). HPV vaccines—A review of the first decade. Gynecol. Oncol..

[84] Fernandes Q., Mestiri S., Bedhiafi T., Raza A., Merhi M., Hydrose S., El-Ella D.M.A., Inchakalody V.P., Uddin S., Dermime S., Duncan L.T. (2022). Chapter 1. Vaccines and Immunotherapies against the Human Papillomavirus and Epstein-Barr Virus. Advances in Health and Disease.

[85] Cuzick J. (2015). Gardasil 9 joins the fight against cervix cancer. Expert Rev. Vaccines.

